# “On the Bat’s Back I Do Fly after Summer Merrily”

**DOI:** 10.3201/eid2907.AC2907

**Published:** 2023-07

**Authors:** Byron Breedlove

**Affiliations:** Centers for Disease Control and Prevention, Atlanta, Georgia, USA

**Keywords:** art science connection, emerging infectious diseases, art and medicine, about the cover, One Health, bats, mammals, fungi, fungal infections, Cryptococcus neoformans, Candida auris, Aspergillus fumigatus, Candida albicans, Biho Takashi, Bat in Moon, On the Bat’s Back I Do Fly after Summer Merrily, Geomyces destructans, Pseudogymnoascus destructans, white-nose syndrome, zoonoses

**Figure:**
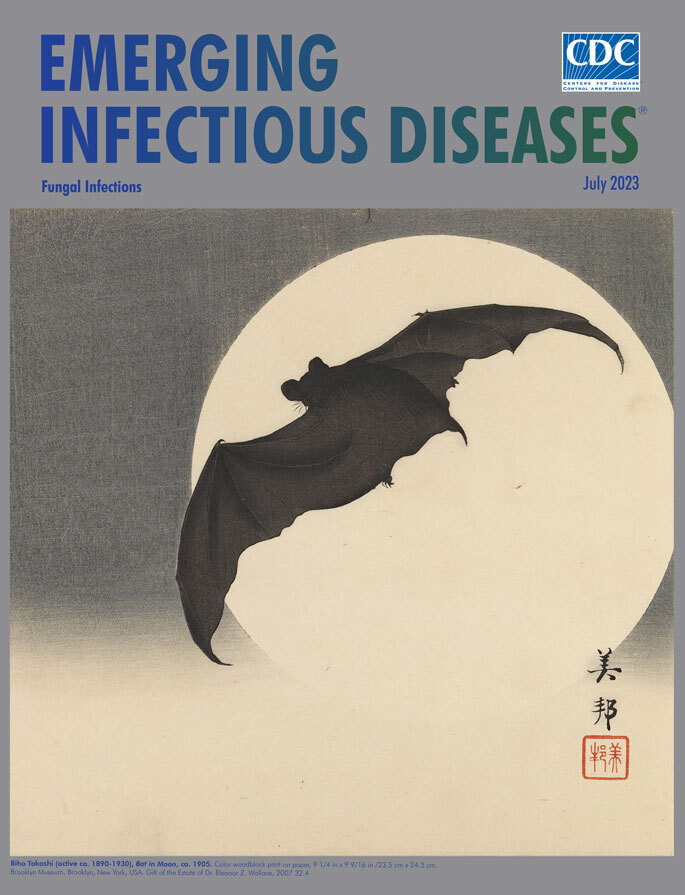
**Biho Takashi (active ca. 1890‒1930). *Bat in Moon*, ca. 1905.** Color woodblock print on paper, 9 1/4 in x 9 9/16 in/23.5 cm x 24.3 cm. Brooklyn Museum, Brooklyn, New York, USA. Gift of the Estate of Dr. Eleanor Z. Wallace, 2007.32.4

The only mammals that can fly, bats play a vital role in maintaining healthy ecosystems. They devour large amounts of insect pests; pollinate hundreds of species of fruit, including avocados, bananas, and mangoes; and spread seeds of a number of plants, including cacao, figs, and nuts. This month’s cover image,* Bat in Moon* by Biho Takashi, portrays a soaring bat silhouetted across a full moon. A fitting caption can be taken from Ariel’s words in Act V, Scene I, of *The Tempest* by William Shakespeare: “On the bat’s back I do fly after summer merrily.” 

Little is known about the artist Biho Takashi. Even his actual name cannot be verified. Woodblocks attributed to Takahashi Bihō or Hirose Bihō bear signatures identical to his. The Five Colleges and Historic Deerfield Museum Consortium in western Massachusetts offers that Biho “was a designer of kachō-e, or ‘animal and flower pictures’ and that he used a gradient effect known as bokashi in many of his works.” 

Biho’s skill with bokashi is revealed by the textures of the bat’s wings, its folded legs visible as creases in the taut wing membranes and its whiskers, claws, wingtips, and tail crisply revealed. The night sky lightens in the bottom third of the image, and its blankness refocuses attention to the bat and moon. The viewer can appreciate the grace and form of an animal that contributes to healthy ecosystems but is also sometimes vilified because of superstition and its role as a host for pathogens that can infect humans. Bats are hosts for a number of viruses that affect humans, and their droppings, when present in large quantity, can potentially infect humans with the fungal disease histoplasmosis.

Researchers James Wynne and Lin-Fa Wang wrote, “In many respects, bats represent the perfect reservoir for emerging zoonotic pathogens. They often live in large colonies or roosts; they can, through flight, travel and disseminate viruses over considerable distances; and they enjoy remarkable longevity for their body size.” However, some of those same attributes that enable bats to be effective reservoirs for viral infections, including their living in large colonies, have had deadly consequences for some bat species.

During the past 2 decades across North America, bat populations have been declining precipitously because of *Pseudogymnoascus* *destructans*, commonly known as white-nose syndrome fungus. Bats infected with this emerging fungus were first detected in the United States in 2006 in a cave frequented by tourists near Albany, New York. Veterinary pathologist Lisa Farina and wildlife pathologist Julie Lankton have stated that an estimated 6 million bats died from this infection within a decade after its discovery and that it “may represent the largest mammalian wildlife mortality event in recorded history.” 

Although this fungus also exists in bats in Europe, it has not proven to cause extensive mortality among those populations. No current evidence suggests this pathogen can infect humans, but circumstantial evidence suggests that, because *P. destructans *survives on shoes, clothing, or gear, humans may have transmitted it to that New York hibernaculum after visiting caves in European locales. 

As the Centers for Disease Control and Prevention notes, “Disease spread by fungi are a One Health issue—fungi that cause human diseases live in the environment and some can spread between animals and people.” Perhaps the appearance of *P. destructans *in bats and* Batrachochytrium dendrobatidis, *a waterborne fungal pathogen that causes chytridiomycosis in amphibians, were bellwether events regarding the emergence of infectious fungal diseases. 

In October 2022, the World Health Organization issued a report stating, “Fungal pathogens are a major threat to public health as they are becoming increasingly common and resistant to treatment with only four classes of antifungal medicines currently available, and few candidates in the clinical pipeline. Most fungal pathogens lack rapid and sensitive diagnostics and those that exist are not widely available or affordable globally.” That report categorized 19 fungal pathogens as priorities and ranked four as critical for public health: *Cryptococcus neoformans*, *Candida auris*, *Aspergillus fumigatus*, and* Candida albicans. *Factors driving the emergence and spread of fungal diseases include climate change, human migration and mass transit, and environmental contamination with antifungal agents. The World Health Organization report explains that among those at highest risk for developing fungal infections are immunocompromised persons, including patients with chronic obstructive pulmonary disease, liver or kidney disease, and viral respiratory tract infections. The report also notes, “The coronavirus disease (COVID-19) pandemic has been associated with an increase in the incidence of comorbid invasive fungal infections.”

Worldwide, fungal infections impose significant healthcare costs and cause substantial morbidity and mortality. In this issue of EID, researchers Jeremy A.W. Gold and colleagues state, “In the United States, fungal infections impose considerable healthcare costs (≈$6.7 billion during 2018) and cause substantial illness and death (>7,000 deaths during 2021).” Other articles document the range and variety of fungal infections affecting humans, including significant increases of severe infections from *C. auris* in healthcare settings in Israel and, from the United States, unusual cases of coccidioidomycosis affecting patient’s ears and incidents of patients who died of fatal invasive mold infections after receiving transplanted organs from drowned donors. Tackling the problems posed by infectious fungal diseases requires public health resources for researchers to develop new antifungal medicines and improve surveillance and diagnostics. Takashi’s *Bat in Moon* serves as an emblematic reminder of the value of scientific research at the One Health intersection of human, animal, and environmental health.
